# Gender differences regarding intention to use mHealth applications in the Dutch elderly population: a cross-sectional study

**DOI:** 10.1186/s12877-022-03130-3

**Published:** 2022-05-24

**Authors:** Floris Ruben Tobias van Elburg, Nicky Sabine Klaver, Anna Petra Nieboer, Marjan Askari

**Affiliations:** 1 Rotterdam School of Management, Erasmus University, Rotterdam, The Netherlands; 2 Erasmus School of Health Policy & Management, Erasmus University Rotterdam, 3000 DR Rotterdam, P.O. Box 1738, Rotterdam, The Netherlands

**Keywords:** Technology Acceptance Model, Intention to use, Older adults, Medical applications, mHealth

## Abstract

**Background:**

In light of the increasing demands in health care, a call has been made for the development of new strategies. One of these strategies is placing a higher emphasis on individuals, who are expected to better manage their own health and illness. mHealth applications could increase this self-management behaviour among older adults. However, it is crucial to know the intention to use mHealth of older adults before implementing these services. Even less is known regarding differences between genders on factors influencing this intention to use mHealth applications.

**Objective:**

The aim of this study was to study the gender differences regarding the relationship between technology acceptance factors and the intention to use mHealth applications in the Dutch elderly population.

**Methods:**

We conducted a quantitative cross-sectional study using questionnaires. The participants were 65 years or older, lived independently or in a senior living facility, without cognitive impairment. Logistic regression with interaction terms was done to determine gender differences in the relationship between the intention to use mHealth applications and technology acceptance factors.

**Results:**

While we found that half of the studied population had intention to use medical applications (50.3%) a notable difference was observed within gender groups which showed more men had intention to use medical applications rather than women (59.4% vs. 43.4% respectively). Adjusted logistic regression analysis per factor on the male and female part of the study population respectively showed that the factors Perceived usefulness (OR 21,69 and 2,39, resp.), Perceived ease of use (OR 7,21 and 2,74), Attitude toward use (OR 24,61 and 4,94), Sense of control (OR 4,12 and 2,67), Personal innovativeness (OR 2,54 and 1,58), Self-perceived effectiveness (OR 3,21 and 2,34), Service availability (OR 4,38 and 2,51) and Facilitating circumstances (OR 3,04 and 2,18) had a statistically significant influence on intention to use in both models. Logistic regression with interaction terms showed that two of the technology acceptance factors differed statistically significant in their relationship with intention to use when comparing females to males, namely Perceived usefulness (OR 0,11) and Attitude toward use (OR 0.24). Both factors were more strongly associated with intention to use for men compared to women.

**Conclusion:**

Policymakers and interventions aiming to stimulate the uptake of mHealth applications should acknowledge gender differences. Interventions based on improving the Perceived usefulness and Attitude toward use among female users could be a means to stimulate the full potential of medical applications and improve the uptake.

**Supplementary Information:**

The online version contains supplementary material available at 10.1186/s12877-022-03130-3.

## Introduction

### Background

Western governments are facing ageing societies, where the number of older adults is expected to triple [1]. Consequences of this global increase are an increase in health care utilization and subsequently additional demand for human resources. In addition, the Dutch health care sector is expected to face a shortage of health care professionals by 2022 [2].

To alleviate the social, societal, and economical pressure that this demographic change creates, governments direct their policies to promote the use of eHealth to increase and support self-management in the form of self-reliance and self-care [3–6]. Current technology trends, growing internet access, and increasing use of mobile applications open avenues for novel services through eHealth and mobile health (mHealth) applications [7, 8]. mHealth can be defined as mobile applications that can gather health information, monitor health and support activities regarding an individual’s health that run on smartphones or tablets [4, 5]. An example of such an app could be a self-measuring and communication tool for blood pressure that alarms the healthcare professional when self-reported values are repeatedly too high. mHealth creates opportunities to deliver new forms of healthcare and to expand services without the need to increase the existing workforce. For example, medical apps have the ability to offer assisted or supervised care and monitoring and enhance the self-management abilities of older adults, which may in turn lead to greater time spent at home and fewer medical visits [3, 9–11].

As older adults are more likely to have multiple chronic diseases [1, 3, 12–14], they are a group that could benefit from the use of medical applications [11, 15–17]. There are challenges for the adoption of mHealth among older adults. Cognitive aging processes, lack of technological skills, physical dexterity, and psychological factors are barriers in the implementation of mHealth for older adults [18]. Studies have also shown that older adults tend to be more resistant in accepting new IT applications [19–21] and possess apprehension toward novel technologies [22]. More recently, several studies have been conducted to advance understanding of the intention to use and actual use of medical technology [23–26]. Our earlier study in the Netherlands showed that almost 50% of the Dutch older adults had no intention to use medical applications [27].

In addition, there are differences in the adoption of technology between males and females [28–31]. Males seem to adopt new technologies more often compared to females since they perceive technology as more useful [32]. This lower tendency of females in adopting technology is severely hindering the full potential use of medical applications. As females have a higher life expectancy [33], they could benefit the most from medical applications. In light of these challenges, it is important to understand the gender differences in the factors influencing the adoption of mHealth in the elderly population.

### Technology acceptance model

One of the most frequently used models for understanding the adoption of technology is the ‘Technology Acceptance Model’ (TAM) which is derived from the Theory of Reasoned Action and the Theory of Planned Behavior [34–36]. The TAM posits that technology adoption is primarily determined by the intention to use a specific type of technology and suggests that factors such as Perceived ease of use and Perceived usefulness influence the intention to use mHealth applications [23, 34, 37]. Additional factors influencing the use of technology complementing the TAM, such as Subjective norm, Image (TAM2), as well as Self-efficacy, Social norms, and Trust were revealed in later studies [3, 10, 25, 38, 39].

### Goal of this study

Earlier studies suggest that gender has an influence on the adoption of mHealth [28–31]. In addition, in view of above mentioned challenges, concepts of the TAM  and all related developed concepts seem to be affected by gender as well. Since men and women possess different traits and societal roles which affect their perceptions and usage of technologies, it is important to study the differences regarding gender. Yet, to the best of our knowledge, no research has been conducted that investigates the differences between gender regarding the relationship between technology acceptance factors and the intention to use mobile medical applications among older adults. Therefore, the aim of this study is to investigate the gender differences regarding intention to use mHealth applications in a quantitative study involving a large sample of older adults.

## Methods

### Study design and data gathering

A cross-sectional study was conducted to understand the differences between genders regarding the relationship between technology acceptance factors and the intention to use medical applications. Data was collected by distributing a questionnaire among Dutch older adults. Our cohort consisted of 360 older adults. The inclusion criteria were seniors aged 65 and above without cognitive impairment who lived independently or in a senior living facility. Respondents were approached in various ways: via elderly living facilities, health care organizations (one hospital, general practitioners and one home care organization), elderly leisure activity clubs and via social media. To increase the accessibility of study participation the questionnaire was distributed both on paper as well as digitally. The reporting of the online questionnaire follows the CHERRIES checklist (Checklist for Reporting Results of Internet E-Surveys), which can be found in Multimedia Appendix 1.

Before participation all participants signed an informed consent form. Thereafter, the data was pseudonymized to ensure anonymity. In the questionnaire, explanation about types of medical applications together with various examples and pictures were provided to the participants. Assistance when filling out the questionnaire and extra explanation was provided to participants by data assistants. The questionnaire was a self-developed instrument composed of various validated measuring instruments. It was checked for quality by five experts (three eHealth experts, one geriatric nurse and one physician). Furthermore, four older adults gave feedback on how to improve the structure and readability. Data was gathered across different regions in the Netherlands for generalizability. The data was entered into a database which was tested for input errors and completeness using four data assistants. A sample of paper-questionnaires was compared to the database counterpart to check if they were identical. Finally, our study was approved by the Medical Ethical Commission of the Erasmus University of Rotterdam under the number MEC-2018–120.

Various demographic data and other background characteristics were gathered, namely sex, age, marital state, education attainment, living situation, quality of life, health literacy and Assessment of Activities of Daily Living, Self-Care, and Independence (ADL) [40]. Quality of life was self-assessed with a one to ten scale where the participants gave a rating to their life. Health literacy was also self-assessed and based on the HLSQM [41]. Assessment of Activities of Daily Living, Self-Care, and Independence (ADL) [40] was calculated using the following calculations. For each ADL item (in total 16 items), the participant answered whether they needed help doing the activity (yes/no). The ADL score was calculated adding all the activities in which no help was needed [40, 42–44]. A summary score ranged from 0 to 16 where the lower the score, the lower the participant’s ability to care for one’s self. In addition, respondents were asked if they had prior experience with the internet and/or mHealth applications.

### Technology acceptance model

The acceptance factors that were used in this study are derived from adapted and expanded versions of the Technology Acceptance Model (TAM). This model suggests that the Perceived ease of use (PEOU) and Perceived usefulness (PU) are key elements in explaining intention to use [34, 36]. Even though TAM has been widely used in the healthcare context, recommendations have been made to integrate additional variables to add more context specificity [45, 46]. Therefore, a number of factors from TAM2 [39], Senior Technology Acceptance Model [23] and the expanded version of Wu (2011) [47] were included. The additional factors that were added were chosen based on a literature review of the deployment of TAM in the context of healthcare and the elderly population. The factors included were Perceived ease of use, Perceived usefulness, Attitude toward use and Subjective norm together with elderly specific factors Sense of control, Anxiety toward use, Personal innovativeness, Social relationships, Self-perceived effectiveness of use, Service availability, Facilitating circumstances and Finance. Descriptions of the TAM variables can be found in multimedia appendix 2. Examples of statements and references for each TAM variable can be found in the study by Askari et al. (2020) [27]. Each TAM factor consists of one to five statements to measure different aspects and strengths of that factor. These statements were answered using a 5-point Likert scale (1 = completely disagree, 2 = disagree, 3 = neutral, 4 = agree, 5 = completely agree). We generated a factor score per TAM factor by calculating the average of the score of all the statements. As stated in the original TAM, intention to use was the outcome variable. To enhance interpretability the outcome variable was transformed to a binary variable. Cases with missing values on any of the TAM variables or control variables were listwise deleted.

The internal consistency of the statements within a factor of the acceptance factors was investigated using Cronbach’s Alpha (CA) [48, 49]. The CA is expressed as a number between 0 and 1. The higher this number is, the lower the error variance is within the measuring instrument. Items that, when removed, increased the Cronbach α of the acceptance factor by 0.1 or more were excluded from the acceptance factor and further analysis. As the Cronbach’s Alpha score within each factor showed an acceptable value above 0.7, all factors were reliable [50, 51].

### Statistical analyses

Descriptive statistics were used to analyze the composition of our sample. For continuous variables, the mean and standard deviation (SD) were calculated and for categorical variables, percentages were used. The demographic characteristics of the two groups (men and women) were compared using the Chi-Square test for nominal variables and the Mann–Whitney U test for continuous variables. If the Chi-Square test was performed with categorical variables, each with two categories, Yates continuity correction was presented, to avoid too small significance values [51].

The generated factor scores of the technology acceptance factors served as input for the multivariate logistic regression analysis to examine the relationship between the intention to use medical applications as the dependent variable and (each of) the technology acceptance factors as independent variables. Age, education, quality of life and ADL served as control variables and, therefore, were always included in the multivariate logistic regression model. The control variables were tested on multicollinearity using a correlation matrix. As none of the control variables had a correlation larger than 0.5 or lower than -0.5 all were included in the logistic regression models [52].

### Analysis of the interaction effects

In addition to a pooled model, separate models for men and women were calculated. OR, coefficient (β) together with *P-*value and standard error (SE) for the factor is reported. To assess whether coefficient estimates differed between genders, an additional pooled model was generated using interaction terms. An interaction occurs if the relationship between the predictor and the outcome is dependent on another variable, in this case gender, and can be tested by adding a product variable to the model [53]. For each of the TAM factors a different interaction term was added into the model. This allowed the slopes of independent variables to change as a function of gender. The *P -*value, standard error and the coefficients (β) of the interaction term are reported.

All the statistical analyses was performed using SPSS Statistics (IBM Corporation, version 25).

## Results

### Population characteristics

Our cohort had an average age of 75 years (SD = 7 years). Out of the 360 participants, 42.6% (*N* = 155) were male. While the majority of the older adults had experience with using the internet, only 16% (*N* = 58) of all participants had experience with medical applications. 50.3% (*N* = 181) of the participants indicated the intention to use such medical applications. The average quality of life was 7.6 (1.2). An overview of other population characteristics can be seen in Table 1.

**Table 1:** Population characteristics in total population and per gender

Characteristics		Total population (360)		Male (*N* = 155) (43.1%)	Female (*N* = 205) (56.9%)	*P -*Value
Age in years, mean (SD)	74.9 (7.0)		74.0 (6.9)		75.6 (7.0)	.020^b^
Education, no. (%)						.004^a^
Lower education	65 (18.7)		22 (14.8)		43 (21.6)	
Intermediate education	159 (45.7)		61 (40.9)		98 (49.2)	
Higher education	124 (35.6)		66 (44.3)		58 (29.2)	
Marital status, no. (%)						< .001^a^
Wedded	189 (52.9)		103 (67.3)		86 (42.2)	
Divorced	51 (14.3)		19 (12.4)		32 (15.7)	
Widowed	87 (24.4)		18 (11.8)		69 (33.8)	
Unwedded	22 (6.2)		7 (4.6)		15 (7.4)	
Sustainable cohabitation	8 (2.2)		6 (3.9)		2 (1.0)	
Living arrangement, no. (%)						< .001^a^
Living independently, alone	126 (35.5)		31 (20.4)		95 (46.8)	
Living independently, with other	160 (45.1)		87 (57.2)		73 (36.0)	
Senior living facility, alone	34 (9.6)		14 (9.2)		20 (9.9)	
Senior living facility, with other	35 (9.9)		20 (13.2)		15 (7.4)	
Health literacy, no. (%)						.267^a c^
Inadequate	49 (14.3)		26 (17.0)		23 (12.2)	
Adequate	293 (85.7)		127 (83.0)		166 (87.8)	
ADL score, mean (SD)	14.7 (2.3)		15.0 (2.2)		14.4 (2.3)	< .001^b^
Quality of life (0–10), mean (SD)	7.6 (1.2)		7.5 (1.3)		7.6 (1.1)	.802^b^
Prior experience with internet, no. (%)	306 (85.2)		143 (92.3)		163 (79.9)	.002^a c^
Prior experience with medical apps, no. (%)	58 (16.2)		26 (16.8)		32 (15.8)	.911^a c^
Intention to use mobile medical apps, no. (%)	181 (50.3)		92 (59.4)		89 (43.4)	.004^a c^

^a^ Chi-Squared test

^b^ Mann–Whitney U test

^c^ Continuity Correction

### Population characteristics per gender

First, the two groups were compared using the Chi-Squared test and the Mann–Whitney U test. As can be seen in Table 1, there are several differences between the groups. Striking differences were the significantly higher prior experience with the internet and the significantly higher intention to use medical applications for males.

### Multivariate analyses per gender

Table 2 shows the pooled logistic regression models for the acceptance factors on intention to use. To give an indication of how values for each TAM variable are spread out, a table with descriptive statistics including mean, standard deviation, median and missing values is provided in Multimedia Appendix 3. Table 3 provides an overview of the results for multivariate analysis per gender group. Table 3 also shows results of multivariate analysis with interaction terms per acceptance factor.

**Table 2:** Odds ratio (OR), standard error (SE) and coefficient estimates of acceptance factors on intention to use

Clusters	POOLED a			
	**OR (95%CI)**	***P* VALUE**	***B***	**S.E**
Gender/female	0.75 (0.46–1.23)	.26	-0.28	.251
Perceived usefulness	4.61 (2.98–7.13)	< .001	1.53	.223
Perceived ease of use	4.09 (2.63–6.37)	< .001	1.41	.226
Attitude toward use	8.31 (4.79–14.43)	< .001	2.12	.282
Subjective norm	1.47 (1.13–1.92)	.005	0.39	.136
Sense of control	3.38 (2.38–4.81)	< .001	1.22	.180
Feelings of anxiety	0.59 (0.44–0.78)	< .001	-0.54	.148
Personal innovativeness	1.94 (1.46–2.59)	< .001	0.67	.147
Social relationship	1.91 (1.19–3.06)	.007	0.65	.240
Self-perceived effectiveness	2.74 (1.90–3.94)	< .001	1.01	.186
Service availability	3.38 (2.25–5.07)	< .001	1.22	.207
Facilitating circumstances	2.52 (1.79–3.55)	< .001	0.92	.175
Finance	1.02 (0.77–1.35)	.92	0.02	.143

^a^ Adjusted for age, education, ADL and quality of life

**Table 3:** Differences in acceptance factors on intention to use between gender groups

Clusters	MALE a				FEMALE a				FEMALE VERSUS MALE a		
	**OR (95%CI)**	***P -value***	***B***	**S.E**	**OR (95%CI)**	***P -value***	***B***	**S.E**	***B***	***P -*value**	**S.E**
Gender/female											
Perceived usefulness	21.69 (6.84–68.81)	< .001	3.08	.589	2.39 (1.48–3.85)	< .001	0.87	.243	-2.19	.001	.628
Perceived ease of use	7.21 (3.18–16.33)	< .001	1.98	.417	2.74 (1.56–4.80)	< .001	1.01	.287	-0.85	.08	.480
Attitude toward use	24.61 (7.23–83.77)	< .001	3.20	.625	4.94 (2.62–9.30)	< .001	1.60	.323	-1.41	.03	.655
Subjective norm	1.49 (0.98–2.25)	.06	0.40	.211	1.43 (0.99–2.08)	.06	0.36	.189	-0.10	.72	.274
Sense of control	4.12 (2.38–7.14)	< .001	1.42	.280	2.67 (1.64–4.35)	< .001	0.98	.248	-0.29	.41	.353
Feelings of anxiety	0.38 (0.23–0.63)	< .001	-0.97	.264	0.76 (0.52–1.12)	.16	-0.27	.196	0.56	.06	.299
Personal innovativeness	2.54 (1.57–4.11)	< .001	0.94	.245	1.58 (1.07–2.33)	.02	0.46	.198	-0.41	.18	.307
Social relationship	2.20 (1.05–4.62)	.04	0.79	.378	1.70 (0.90–3.23)	.10	0.53	.327	-0.27	.57	.479
Self-perceived effectiveness	3.21 (1.82–5.66)	< .001	1.17	.289	2.34 (1.42–3.86)	.001	0.85	.255	-0.29	.44	.372
Service availability	4.38 (2.28–8.41)	< .001	1.48	.333	2.51 (1.48–4.24)	.001	0.92	.268	-0.55	.19	.415
Facilitating circumstances	3.04 (1.81–5.11)	< .001	1.11	0.27	2.18 (1.34–3.56)	.002	0.78	.250	-0.30	.39	.347
Finance	0.92 (0.59–1.42)	.69	-0.09	.225	1.04 (0.71–1.53)	.85	0.04	.196	0.18	.53	.286

^a^ Adjusted for age, education, ADL and quality of life

In the pooled model, all factors except Finance were found to be significantly related to the intention to use, in accordance with what we reported in an earlier article [27]. All factors had a positive association with intention to use, except for Feelings of anxiety, which had a negative association meaning that higher levels of anxiety were associated with lower intention to use.

With three exceptions, all factors that were found significant in the pooled model remained significant in the male and female model. Subjective norm was not significant in the male and female model, and Social relationships and Feelings of anxiety were significant in the male model, but not significant in the female model. Striking is that all factors, with the exception of Feelings of anxiety and Finance, showed higher odds ratios in the male only model compared to the female model. In the model with interaction terms, only the interaction term for Perceived usefulness and Attitude toward use were significant. Both factors showed a stronger relationship with intention to use for males compared to females.

## Discussion

### Principal results

This study was performed to investigate possible gender differences in the relationship of technology acceptance factors and the intention to use medical applications among older adults. To do so, technology acceptance factors were explored that have potential for policy makers and care givers to act on in the pursuit of a greater uptake of mHealth among older adults. While we found that half of the studied population had intention to use medical applications (50.3%) a notable difference was observed within gender groups which showed more men had the intention to use medical applications rather than women (59.4% vs. 43.4% respectively). In addition, men had more prior experience with internet (92.3% vs. 79.9% respectively men vs. women), which could possibly be related to a higher intention to use. This first finding is supported by previous research [54], and could be explained by the social role of men, being more adventurous and more open to try new things when it comes to technology [29].

We used a wide variety of sources to collect our data (a hospital, general practitioners offices, elderly leisure activity clubs) in various regions across the Netherlands, using both online and paper questionnaires. These efforts were all aimed at creating a representative sample contributing to generalizability of the findings. In addition, our population had comparable quality of life and ADL scores as the general population in the Netherlands [55, 56]. However, the intention to use medical applications might be different in an elderly population where (multiple) chronic diseases are more prevalent [57, 58]. Therefore, future study needs to distinguish this particular group also because on average they are likely to be older and may need other policies to increase mHealth uptake.

Our results showed there was a significant difference between male and female groups regarding two factors: Perceived usefulness and Attitude toward use. Perceived usefulness was more strongly linked to intention to use for men than for women. A possible explanation for this is that men are primarily focused on practical purposes and the accomplishment of goals, and therefore more concerned with the usefulness of new technologies [29, 36, 59]. Having positive attitudes toward the use of technology was also a more strong predictor for the intention to use medical applications for elderly men compared to women. This finding is supported by earlier research which showed that females in general have a lower Attitude toward technology than males [30, 60]. Cai et al. (2017) state that the scarce participation of women in technology, based on the general view that technology is a male dominated area, could be one of the explaining factors for their lower attitude because these social prejudgments could form a barrier for women to gather interest about technology. There are studies showing that women are more concerned with their health, more active in seeking health-related information and seek help of healthcare professionals more early compared to men [61–63]. As our results showed that females are less likely to adopt technology for the good of their own health, this points to contradictory results regarding mHealth as compared to these other health behaviors suggesting other policies are needed for mHealth implementation among women to increase the uptake and strengthen long-lasting adoption. In addition, we found that the factors Attitude toward use, Sense of control, Personal innovativeness, Self-perceived effectiveness, Service availability and Facilitating circumstances were relevant for both the female and male groups. These findings are relevant when placing them in the context of facilitating the uptake of medical applications among older adults. Medical applications can contribute in the delivery of long-term care for chronic diseases [64, 65]. This is relevant for older adults, because of the high prevalence of these diseases in that population [65].

An argument could be made that older adults who live alone (e.g. without a partner) could benefit even more from this technology, as their need for self-management is even greater. Elderly women live alone more often than their male counterparts [66], hence the need for mHealth solutions might be greater for women. Additionally, our findings suggest that females are lagging behind in the use of medical applications when compared to males. This suggests that to increase the uptake of mHealth among older adults, females might be the group to consider first as the greatest potential for an increase in uptake lies within this group. In accordance, our findings suggest that the uptake of mHealth applications could be stimulated by putting an emphasis on the needs of women while creating policy and interventions.

### Comparison with prior work

To the best of our knowledge, this is the first study investigating the differences in gender regarding influencing acceptance factors among older adults and therefore comparison which earlier studies might be difficult. However, in the study done by Faqih et al. in 2015, the influence of gender on the relationship between TAM factors and behavioral intention regarding mobile health was investigated not specifically among older adults [29]. In this research they studied several hypotheses of which two are of interest: 1) ‘Perceived usefulness’ influences behavioral intention to adopt mHealth more strongly for men than women; and 2) ‘Perceived ease of use’ influences behavioral intention to adopt mHealth more strongly for women than men. Their results showed that hypothesis one was not supported, however hypothesis two was supported.

Their results are not in line with our results for older men and women. Our analyses showed that the ‘Perceived usefulness’ influenced the intention to use medical applications more strongly for older men compared to older women. In addition, we found that Perceived ease of use did not have a stronger effect on the intention to use for older women compared to older men. Still, other studies in non-healthcare domains have reported findings in harmony with our study results [36, 67, 68].

Although the population size of Faqih and Riad Mousa Jaradat (2015) is almost identical to ours, there are some significant differences to be found, possibly explaining the difference in results. First, their population does not consist of older adults. Only 4.6% of their respondents was over 50 years of age. Since age had a moderating effect on the relationships between TAM-factors and intention to use, it is not possible to draw fully accurate comparisons between our study and the study of Faqih and Riad Mousa Jaradat, (2015). Second, a large part of the population of Faqih and Riad Mousa Jaradat (2015) consists of participants with a fairly high educational level (65% of the participants have either a bachelor’s degree or a bachelor’s and a master’s degree). It can be argued that due to the relatively young and highly educated population the overall tech-savviness is expected to be higher. This in turn can greatly influence both the behavioral intention, in our case intention to use, as well as what is perceived as useful.

For example, their second hypothesis can be explained through social role division. Women tend to be more concerned with ease of use, rather than the accomplishment of tasks when using a new technology [36, 69]. Other empirical results support that women are highly motivated by Perceived ease of use, although none of these studies looked specifically at the elderly population [67, 68]. A possible explanation for this difference is that this social role division is less prominent in the older generations. Among the elderly, both genders are evenly unexperienced with regard to new technology because they were not raised with such technology. Therefore, both genders face the same difficulties in ease of use of a new technology.

### Limitations

Our study also has some limitations. In the current study we only used “male” and “female” as answering options. Even though we did not have missing values, future research should include a category for “others” given that the gender discussion is much broader than male versus female.

Another limitation was, that the questionnaire used to collect the data was rather elaborate. Although steps were taken to minimize the impact of the length of the questionnaire, such as printing out the questionnaire so participants could take breaks or sitting with the respondents while they filled out the questionnaire, some participants still showed signs of response-fatigue. Respondent fatigue is a well-documented phenomenon that occurs when survey participants become tired of the survey task and the quality of the data they provide begins to deteriorate. It occurs when survey participants' attention and motivation drop toward later sections of a questionnaire [70].

We noticed that some of the participants, especially the group with an age above 75, struggled with grasping what medical applications fundamentally are, how they work, how they could be used, etc. Although, this might result in situations where participants’ answers might not fully reflect their actual opinion, we tried to provide additional explanation about medical applications both within the questionnaire, verbally or by means of demonstration when this was necessary in addition to the written explanation and pictures in the questionnaire.

Our study had a cross-sectional design. Therefore, no claims of causality can be made [71] and the results might suffer from self-report bias [72]. Longitudinal studies are needed to investigate the causal relationship between gender and acceptance factors. Further investigation could study the mechanisms underlying the acceptance factors influencing the intention to use. In addition, some differences between this study and the current available literature were found. Although the differences could be attributed to the differences between the population under study, future research should validate the proposed study results in other elderly populations.

Lastly, this study is based on TAM and several adapted versions of this model. A critical point can be made that the frequent adaption has weakened the model and distanced it from the theory it was originally based on [44]. Although TAM in its original form can have a high explained variance in technology adoption, researchers call for more context specificity, especially in the healthcare context [44]. TAM must also be adapted to study relatively new technology like mobile applications [73]. Therefore, extensive literature research and careful selection of additional factors was done to facilitate an as-complete-as-possible analysis of a set of related factors of technology adoption among the elderly. As described in an earlier paper (Askari et al., 2020), high odds ratios and explained variance indicate that the most important factors associated with the intention to use medical apps have been identified supporting content validity of the measurement instrument.

### Implication and recommendations

This study provided specific areas of focus for policy makers and care/software providers to take into account when facing problems on the uptake of medical applications. This study can recommend that policy based on technology acceptance factors should acknowledge the gender difference. Since elderly females are the group that is suggested to be the most difficult to reach, and at the same time the group that could benefit from mHealth interventions the most, the adoption factors that best define the difference in intention to use should be taken into account when creating interventions. Perceived ease of use, Sense of control, Personal innovativeness, Self-perceived effectiveness, Service availability and Facilitating circumstances are a good means to stimulate mHealth uptake among the general elderly population. Interventions based on Perceived usefulness and Attitude towards use are more appropriate when targeting elderly males. Therefore, if the aim of policy is to stimulate mHealth use among females, other factors should be considered first.

The gender gap in technology use is already closing due to educational and social developments [59]. Policymakers could make use of this existing movement. This requires traditional ways of thinking about social role division with regard to technology use to be disregarded. An example of this could be interventions that pose to increase awareness of the benefits and the added value of medical applications. In addition, interventions that improve trust in mHealth could help to increase the appreciation of the usefulness of medical applications among women [29]. These interventions might entail promoting the usefulness and necessity of medical applications as well as training the elderly females on functionalities of the application. Education has been proven to be an effective way to stimulate positive attitudes toward technology use among women [29]. Training and providing information could be specifically targeted at women by including it as a standard part of a treatment for chronic conditions that affect relatively more women than men.

## Supplementary Information

**Additional file 1: Multimedia Appendix 1. **CHERRIES checklist.

**Additional file 2: Multimedia Appendix 2. **Description of TAM variables.

**Additional file 3: Multimedia Appendix 3.** Descriptive statistics of TAM variables.

## Data Availability

The datasets generated during and analyzed during the current study are not publicly available due to unpublished/ongoing studies but are available from the corresponding author on reasonable request.

## Abbreviations

OR: Odds ratio
TAM: Technology acceptance model
