# Supplementary material for: Gender differences regarding intention to use mHealth applications in the Dutch elderly population: a cross-sectional study
Source: BMC Geriatr. 2022 May 24;22:449. doi: 10.1186/s12877-022-03130-3 (PMC9128125; doi:10.1186/s12877-022-03130-3)
Supplement: Supplementary file 2 — Additional file 2: Multimedia Appendix 2. Description of TAM variables. [file 12877_2022_3130_MOESM2_ESM.docx]

## Multimedia Appendix 2: Description of TAM variables

| Perceived usefulness | The extent to which a person believes that using the medical app will improve his or her quality of life |
| --- | --- |
| Perceived ease of use | The extent to which a person believes that using medical apps will be free of effort |
| Attitude toward use | An individual’s positive or negative feelings or appraisal about using medical apps |
| Subjective norm | The person’s perception that most people who are important to them think they should or should not use medical apps |
| Sense of control | The perceptions of internal and external constraints on using medical apps |
| Feelings of anxiety | An individual’s apprehension when he or she is faced with the possibility of using technology |
| Personal innovativeness | Personal tendency to innovate, or introduce something new or different |
| Social relationship | An individual’s satisfaction with personal relationships and support from friends and family |
| Self-perceived effectiveness | Judgment of one’s ability to use medical apps to accomplish a particular job or task |
| Service availability | The obtainability and accessibility of medical apps |
| Facilitating circumstances | Objective factors in the environment that can make technology usage easy. Included indicators are basic knowledge and available help |
| Finance | Having the financial resources to make technology usage easy |
