# Supplementary material for: Gender differences regarding intention to use mHealth applications in the Dutch elderly population: a cross-sectional study
Source: BMC Geriatr. 2022 May 24;22:449. doi: 10.1186/s12877-022-03130-3 (PMC9128125; doi:10.1186/s12877-022-03130-3)
Supplement: Supplementary file 3 — Additional file 3: Multimedia Appendix 3. Descriptive statistics of TAM variables. [file 12877_2022_3130_MOESM3_ESM.docx]

## Multimedia Appendix 3: Descriptive statistics of TAM variables

| Variable | Mean (95% CI) | Standard deviation | Median | Missing n (%) |
| --- | --- | --- | --- | --- |
| Perceived usefulness | 3.02 (3.10 – 3.31) | 0.98 | 3.33 | 42 (11.7) |
| Perceived ease of use | 3.07 (2.96 – 3.17) | 0.93 | 3.00 | 52 (14.4) |
| Attitude | 3.25 (3.15 – 3.36) | 0.97 | 3.25 | 43 (11.9) |
| Subjective norm | 2.43 (2.32 – 2.54) | 1.01 | 3.00 | 39 (10.8) |
| Sense of control | 2.98 (2.86 – 3.10) | 1.08 | 3.00 | 44 (12.2) |
| Feelings of anxiety | 2.69 (2.58 – 2.80) | 1.04 | 3.00 | 35 (9.7) |
| Personal innovativeness | 2.64 (2.53 – 2.75) | 1.04 | 2.75 | 36 (10.0) |
| Social relationships | 4.06 (3.99 – 4.13) | 0.62 | 4.00 | 26 (7.2) |
| Self-perceived effectiveness | 3.25 (3.14 – 3.36) | 0.98 | 3.50 | 37 (10.3) |
| Service availability | 2.97 (2.86 – 3.07) | 0.94 | 3.00 | 47 (13.3) |
| Facilitating circumstances | 3.06 (2.95 – 3.16) | 0.96 | 3.00 | 42 (11.7) |
| Finance | 2.43 (2.32 – 2.54) | 0.98 | 2.00 | 41 (11.4) |
